# Pathological correlations between traumatic brain injury and chronic neurodegenerative diseases

**DOI:** 10.1186/s40035-017-0088-2

**Published:** 2017-07-11

**Authors:** Marcela Cruz-Haces, Jonathan Tang, Glen Acosta, Joseph Fernandez, Riyi Shi

**Affiliations:** 1 Weldon School of Biomedical Engineering, Purdue University, West Lafayette, IN 47907 USA; 2 Department of Basic Medical Sciences, Purdue University, West Lafayette, USA

**Keywords:** Traumatic brain injury, Neurodegenerative diseases, Alzheimer’s disease, Parkinson’s disease, Amyotrophic lateral sclerosis, Oxidative stress, Reactive oxygen species

## Abstract

Traumatic brain injury is among the most common causes of death and disability in youth and young adults. In addition to the acute risk of morbidity with moderate to severe injuries, traumatic brain injury is associated with a number of chronic neurological and neuropsychiatric sequelae including neurodegenerative diseases such as Alzheimer’s disease and Parkinson’s disease. However, despite the high incidence of traumatic brain injuries and the established clinical correlation with neurodegeneration, the causative factors linking these processes have not yet been fully elucidated. Apart from removal from activity, few, if any prophylactic treatments against post-traumatic brain injury neurodegeneration exist. Therefore, it is imperative to understand the pathophysiological mechanisms of traumatic brain injury and neurodegeneration in order to identify potential factors that initiate neurodegenerative processes. Oxidative stress, neuroinflammation, and glutamatergic excitotoxicity have previously been implicated in both secondary brain injury and neurodegeneration. In particular, reactive oxygen species appear to be key in mediating molecular insult in neuroinflammation and excitotoxicity. As such, it is likely that post injury oxidative stress is a key mechanism which links traumatic brain injury to increased risk of neurodegeneration. Consequently, reactive oxygen species and their subsequent byproducts may serve as novel fluid markers for identification and monitoring of cellular damage. Furthermore, these reactive species may further serve as a suitable therapeutic target to reduce the risk of post-injury neurodegeneration and provide long term quality of life improvements for those suffering from traumatic brain injury.

## Background

Traumatic brain injury (TBI) represents one of the most common causes of death and disability in young people [1–3]. About 1.6 million people per year experience traumatic brain injuries in the USA [4, 5]. Besides the initial mechanical damage, TBI can induce a process of secondary injury [6, 7], which can lead to long term neurological and neuropsychiatric sequelae [8, 9], depicting a serious public health problem worldwide [10]. Some of the observed post-TBI sequelae include, but are not limited to, neurodegenerative diseases [11], such as Alzheimer’s disease (AD) [12], Parkinson’s disease (PD) [13], and amyotrophic lateral sclerosis (ALS) [14].

Importantly, the mechanisms underlying the pathogenesis that lead to such disabilities are still incompletely understood [15, 16]. Therefore, while the post-TBI central nervous system (CNS) illnesses have a high prevalence [17]; few, if any, treatments are available to deter and prevent the pathological progression thought to lead to chronic neurological diseases and conditions [18–21]. Thus, a better understanding of the molecular mechanisms underlying TBI and neurological diseases is crucial to uncover the potential link between these conditions to enable development of effective diagnostic and treatment strategies which could reduce the incidence of post-TBI neurological complications.

This review intends to present the analysis of the current related published literature, that could lead to a better understanding of the mechanisms underlying TBI and neurodegenerative diseases, that might be linked to the development of neurodegenerative diseases post-TBI.

## Pathological mechanisms of TBI

In most cases, TBI results from a physical blow to the head during traumatic events such as falls [22], motor vehicle collisions [23], or sports related injuries [24], although these injuries can also be inflicted by exposure to explosive blasts [25]. TBI is currently classified as mild, moderate, or severe based on clinical observations and history such as duration of loss of consciousness and post traumatic amnesia [26, 27]. Mild TBI (mTBI) comprises the majority of cases [28]; however, diagnosis is primarily by exclusion of injuries requiring specific intervention [29]. Furthermore, inconsistent clinical definitions between governing organizations presents challenges in comparing incidence rates of mTBI [28, 30]. This difficulty in diagnosis can be a serious concern due to acute effects such as second impact syndrome [24] or through chronic effects arising from repetitive TBI [31].

Damage to nervous tissue can be classified as primary injury, which occurs as a direct result of the experienced physical forces [32]; and secondary injury, which arises from pathophysiological processes following the traumatic event [33]. The primary injury process consists of the rapid acceleration-deceleration applied to the head, which is thought to damage the brain by producing shear forces within nervous tissue resulting in axonal injury and impact with the cranial wall [34]. These injuries can be ipsilateral or contralateral to the blow, and have been described in literature as coup and contre-coup, respectively [35]. In more severe cases, injury can cause intracranial hemorrhage and subsequent intracranial hypertension [26]. This increase in pressure not only damages brain tissue by compression, but also by causing cerebral hypoperfusion and potential ischemic injury by decreasing cerebral perfusion pressure [36].

Secondary injury in TBI typically occurs in the days, weeks, and months following the traumatic event due to biochemical changes in nervous tissue [37, 38]. This damage is frequently mediated by free radicals and reactive oxygen species (ROS) produced from ischemia-reperfusion injury, glutamatergic excitotoxicity, or neuroinflammation [39–41]. Following the initial trauma, axonal damage from the shear forces of primary injury affects membrane permeability and ionic balance [42]. In particular, uptake of calcium through either membrane disruption or activation of NMDA and AMPA receptors by glutamate can result in mitochondrial dysfunction and overproduction of free radicals and activation of apoptotic caspase signaling [43–45]. Subsequent inflammatory processes such as activation of native microglia may also contribute to oxidative stress via oxidative burst or through secondary effects of inflammatory cytokines [46]. These reactive radicals can overwhelm endogenous antioxidant systems and inflict cellular damage via lipid peroxidation and protein modifications [47]. The secondary products of free radical mediated lipid peroxidation, such as reactive carbonyl species, are also electrophilic and can further propagate oxidative damage to biomacromolecules [48, 49].

Clinical and preclinical studies have demonstrated the presence of oxidative stress and its byproducts following TBI with both serological and histological methods [50–52]. In animal studies, these products have been shown to be elevated as early as one day [53] following a single traumatic event and to persist up to 42 days with repeated injury [50]. Furthermore, spectroscopic evaluation suggests that the major endogenous antioxidants glutathione and ascorbic acid may remain diminished for 3 and 14 days post injury, respectively [38]. Elevation of F_2_-isoprostane, a lipid peroxidation byproduct, has been observed in the cerebrospinal fluid of human severe TBI patients with peak levels at 1 day post injury; however this was primarily an evaluation of hypothermia treatment and did not establish comparison with healthy controls [47]. Lipid peroxidation products such as 4-hydroxynoneal were also found to be elevated in the serum of severe TBI patients requiring long term care [54]. Although chronic oxidative stress has not currently been observed following single mild injuries in humans, it appears likely that oxidative stress and its associated processes may exacerbate or prolong post-concussive symptoms [55]. Given the common involvement of oxidative stress in excitotoxicity and reperfusion injury, it is likely that oxidative stress plays a central role in secondary neuronal injury following TBI.

The pathological mechanisms in secondary TBI are particularly interesting due to capacity to prolong cellular injury beyond the initial traumatic event. Some of these characteristic changes, such as oxidative stress and excitotoxicity, have also been observed in the pathophysiology of neurodegenerative diseases which suggests a potential pathological mechanistic link between TBI and neurodegenerative diseases. Therefore, review of the pathological mechanisms in neurodegenerative diseases and TBI may be helpful in elucidating the causative factors for development of neurodegenerative diseases after TBI.

## Pathological mechanisms of neurodegenerative diseases

Despite divergent clinical presentation, AD, PD, and ALS have several common characteristics [56]. Each disease has identified genetic risk factors, although most cases are idiopathic [57–59]. Pathologically, these diseases are characterized by the degeneration of specific neuronal populations associated with the observed clinical symptoms [60, 61]. In addition, aggregation or dysfunction of amyloid-β (Aβ), α-synuclein, and superoxide dismutase (SOD1) are commonly found in AD, PD, and ALS, respectively [56, 62–65]. Although the exact mechanisms of pathogenesis have not been fully determined, it has been suggested that oxidative stress, glutamatergic excitotoxicity, and neuroinflammation play key roles in the pathophysiology of neurodegeneration, particularly in AD [66–71], PD [72–76], and ALS [56, 77–81].

Alzheimer’s disease has an extraordinary high prevalence in the elderly population that greatly reduces the quality of life and the survival [82]. In 2008, as many people as 24 million had dementia world-wide, of whom most had AD; number which was expected to double every 20 years as the population aged [83]. Alzheimer’s disease’s pathology involves the presence of neuritic plaques and the loss of cholinergic neurons in the brain, but the underlying mechanisms leading to these events are still unclear [84]. Neurodegeneration in Alzheimer’s disease is thought to occur due to the accumulation of amyloid β-peptide (Aβ) in plaques in the brain tissue, but the mechanisms underlying its aggregation and toxicity are still incompletely understood [85].

Specifically, studies have indicated that oxidative stress might play a major role in AD pathogenesis [86], due to direct evidence of increased neurotoxic markers of lipid peroxidation, such as 4-hydroxynonenal, in human subjects [87], excessive brain protein oxidation in AD [88], increased nuclear DNA oxidation in the brain of AD patients [89], 30% increased activity of the free radical scavenging enzyme SOD-1 in cell lines of AD patients [90], and significantly, direct evidence that beta amyloid generates free radical peptides [70, 91]. Additionally, it has been well documented that Aβ-induced free radicals and lipid peroxidation are major contributors to neuronal death in AD [92, 93]. Remarkably, in vitro and animal studies have shown that the antioxidant effect of cannabinoids was able to prevent the neurodegenerative process occurring in the disease [94], suggestive of the important role of oxidative stress in the neurodegenerative process of AD.

Another process that has been related to Aβ toxicity is inflammation, which has also been linked to oxidative stress by inflammatory cytokines’ activity [95]. Under healthy conditions, inflammatory processes are expected to restore cellular homeostasis and rebalance redox equilibrium [96]; but in AD conditions, the inflammatory processes are altered with co-localized Aβ deposits, inflammatory related proteins, and activated microglia [97]. Microglia and astroglia recognize misfolded and aggregated proteins to trigger an innate immune response that contributes to the disease progression and severity [98]. In other words, microglia recruitment promotes Aβ clearance and is neuroprotective in early stages of AD, but as the disease progresses, inflammatory cytokines downregulate Aβ clearance genes, and promote Aβ accumulation, contributing to neurodegeneration [99]. Furthermore, cytokines can induce the production of arachidonic acid, which exacerbates neurodegenerative processes by increasing extracellular levels of glutamate, known to cause excitotoxicity in AD [100]; and can also lead to the formation of superoxide free radicals, which have a direct effect in cellular death [101]. Moreover, studies suggest that non-enzymatically glycated tau induces oxidative stress, which results in cytokine gene expression and release of Aβ-peptide in AD [102], indicating a vicious pathological mechanistic circle between cytokines and oxidative stress that contributes to the progression and severity of AD. Additionally, oxidative damage from reactive oxygen species and lipid peroxidation products, such as 4-hydroxy-2-nonenal (HNE), is capable of inhibiting glutamate transporters, causing a decreased glutamate uptake critical for neuronal survival, an increased glutamate concentration in the synaptic cleft, and subsequent excitotoxicity that leads to neurodegeneration in AD [103].

Chronic traumatic encephalopathy (CTE) is a progressive neurodegenerative syndrome associated with repeated blunt force impacts to the head with transfer of acceleration and deceleration forces to the brain [104]; in other words, caused by repetitive mild traumatic brain injuries [105], although the central pathological mechanism explaining the development of progressive degeneration in CTE has not been elucidated [106]. CTE has been clinically associated with behavioral and personality changes, parkinsonism and dementia [107, 108]. Original findings of CTE studies were similar to Alzheimer’s disease, but different in the predominance of tau protein deposition over amyloid [109]. The tau protein deposition in CTE is interesting since it has been previously demonstrated that tau inhibits kinesin-dependent transport of peroxisomes, and that the loss of peroxisomes makes the cells vulnerable to oxidative stress, leading to degeneration [110]. This tau protein deposition, which also occurs in AD but less dramatically when compared to CTE, also inhibits the transport of amyloid precursor protein (APP) into axons or dendrites, causing its accumulation in the cell body [110]. Besides tau proteins, fragments of TDP43, a nuclear RNA/DNA binding protein that regulates the transcription of thousands of genes [111], have been identified in AD, PD, ALS, and CTE, which induce the misfolding of SOD1, predisposing the surrounding cells to free-radical damage [112, 113]. These observations indicate the relevance of oxidative stress also in CTE neurodegeneration.

Chronic inflammation has also been observed in CTE and AD, which is thought to exacerbate the neurodegenerative process [31], and as previously described, has a relationship with oxidative stress though inflammatory cytokines. Moreover, it has been previously described that after the initial head trauma in CTE, microglia get activated and release toxic levels of cytokines, excitotoxins like glutamate, etc.; the excitotoxins inhibit phosphatases, resulting in hyperphosphorylated tau, neurotubule dysfunction, and neurofibrillary tangle deposition, all being relevant components of the CTE syndrome; and besides, there appears to be a synergy between proinflammatory cytokines and glutamate receptors that increases reactive oxygen species and worsens neurodegeneration in the injured brain [106, 114].

Parkinson’s disease is the second most prevalent neurodegenerative disease in industrialized countries with prevalence of approximately 0.3% of the adult population [59]. Histologically, PD is characterized by the formation of α-synuclein rich Lewy bodies and subsequent death of the dopaminergic neurons of the *substantia nigra* [63]. Several genetic risk factors have been identified including mutations to the ubiquitin proteasome system [59, 115]. Although the exact mechanisms which initiate dopaminergic degeneration in non-hereditary PD are still unclear, it has been suggested that oxidative modification or carbonylation of the lysine rich N-terminus and non-amyloid component of α-synuclein may contribute to α-synuclein aggregation [63, 116, 117].

Consistent with this notion, the reactive carbonyls produced as secondary products in oxidative stress have been shown to form lysine adducts and induce α-synuclein aggregation in vitro [118, 119]. In addition, animal models of PD using agents such as 1-methyl-4-phenyl-1,2,3,6-tetrahydropyridine have demonstrated increased production of superoxide in dopaminergic cells relative to cortex [120]. Furthermore, mitochondrial localization of α-synuclein has been shown to promote oxidative stress in vitro [121]. Neuroinflammation has been proposed as a partial contributor to the oxidative stress in PD [122] with activated microglia being observed in the *substantia nigra* and striatum of deceased PD patients [123, 124]. Similarly, activated microglia were seen in rhesus monkeys up to 14 years after model induction [125]. Additionally, glutamatergic excitotoxicity has been proposed to play a role in PD. Rotigotine, an FDA approved dopamine receptor agonist, has been suggested to improve the efficiency of glutamate transporter 1 [126] (GLT-1), and has been shown to offer neuroprotection against glutamatergic excitotoxicity in dopaminergic cell culture [127].

On the other hand, ALS is a fatal neurodegenerative disease characterized by the death of motor neurons in the central nervous system and is the most common motor neuron disease [128]. Approximately 10% of ALS cases have been attributed to genetic causes while the majority are idiopathic [57]. Mutations affecting superoxide dismutase (SOD1) account for nearly 20%, of familial cases; however, this accounts for only 2% of cases overall [58]. Despite identification of these mutations, the exact pathological mechanism is yet to be determined [129].

SOD1 mutant mouse models have demonstrated formation of SOD1 aggregates [64]. Given the role of SOD1 in detoxification of the superoxide radical [130], it was previously suggested that loss of function could cause increased cellular exposure to reactive oxygen species [131]; however, this hypothesis has been challenged by findings of normal development of SOD1 deficient mice in the absence of significant traumatic insult [132]. Furthermore, Bruijn et al. found that SOD1 mutant animals showed no significant improvement in symptomatic progression with knockout or coexpression of wild type SOD1 [64] which suggests that the mutation results not in loss of function, but rather a gain of toxic properties. Studies in rats and human patients suggest that, similar to α-synuclein and Aβ, SOD1 mutation results in formation of potentially cytotoxic protein aggregates even in patients lacking known mutations of SOD1 [62, 64, 133]. In addition, the altered catalysis performed by some mutant variants results in diminished astroglial reuptake of glutamate via inhibition of GLT-1 [134–137]. Indeed, Riluzole, an FDA approved treatment for ALS, has been suggested to alleviate glutamatergic excitotoxicity via a variety of mechanisms including increased glutamate uptake via GLT-1 [138] and blockade of sensitive channels [126]. Hence, it appears that oxidative stress is also involved in the processes of neuronal death and disease progression in ALS [139].

Given its role in mediating damage from neuroinflammation and excitotoxicity, it is likely that oxidative stress plays an important role in the pathophysiology of AD, PD, and ALS in a similar fashion to TBI. As such, addressing oxidative stress in neurodegeneration could serve as an effective strategy in neuroprotection.

## Behavioral and molecular mechanisms linking TBI to neurodegenerative diseases

Several studies have reported an increased incidence of the development of neurodegenerative diseases after TBI events. Previous reports have indicated a three times higher incidence of PD among TBI victims, compared to overall cases [13]. Similarly, the incidence of AD has been reported to be higher for post-TBI cases [140, 141]. TBI has also been suggested to be a risk factor for ALS with repeated studies in professional Italian soccer players showing elevated risk of disease [142, 143]. A case control study of ALS patients in the United States also found a nearly 11-fold increase in ALS risk with repeated TBI [14]. However, at this time it appears unlikely that a single occurrence of TBI significantly affects risk of ALS [14, 144]. In addition, chronic traumatic encephalitis (CTE), a tau pathology, has drawn increasing attention due to presence in NFL players and professional athletes that suffer from repeated TBI [107, 145]. Because the incidence of neurodegenerative diseases and conditions appears to be increased after TBI, it is relevant to discuss the possible behavioral and molecular mechanisms linking TBI to neurodegeneration.

TBI victims and TBI animal models have been shown to present characteristic pathological changes in key proteins, reflecting the interruption of axonal transport due to axonal injury [146]. The accumulated proteins that induce protein neuropathy include Aβ [147], α-synuclein [148], and tau protein [149]. These protein changes are particularly interesting, since it is well-established that Aβ protein aggregation is an important pathological component of AD [150], α-synuclein protein aggregation is a critical characteristic of PD [151, 152], and tau protein aggregation is important in the pathogenesis of CTE [153] and AD [154]. Remarkably, these protein neuropathological changes can be promoted in all three proteins via oxidative stress related free radicals and reactive aldehydes which are commonly elevated following TBI [77, 118, 119, 155, 156]. In addition, the reactive aldehyde byproducts of lipid peroxidation have been shown to cause further lipid peroxidation [52]. Given that these pathological protein states can also induce production of free radicals through excitotoxicity [127, 128] or alteration of mitochondrial ion balance [92, 121] and that reactive aldehydes can induce further lipid peroxidation and protein carbonylation [48, 49, 157, 158], it is possible that oxidative stress holds a key role in a self-propagating cycle of lipid peroxidation, protein carbonylation, and neurodegenerative protein aggregation.

TBI patients and TBI animal models have shown behavioral signs such as post-TBI dementia that resembles AD [159]; post-TBI motor deficits that provide evidence of post-TBI brain tissue damage in the area of the hippocampus [160], resembling brain tissue damage in AD [161]; and damage in the basal ganglia [162], resembling the brain tissue damage that occurs in PD [163]. Functional magnetic resonance imaging (fMRI) studies have also indicated transient and persistent neuropathological functional changes in the brain of TBI victims that could contribute to the development of chronic neurodegenerative diseases [164]. These changes observed in post-injury patients suggest that TBI could inflict the initial tissue damage that resembles or promotes processes common in the pathophysiology of neurodegenerative diseases.

Based on the central role that oxidative stress plays in post-TBI secondary injury and in the pathophysiology of neurodegeneration, it is probable that oxidative stress is a key process in linking TBI to increased incidence of neurodegeneration. Therefore, oxidative stress may serve as a therapeutic, diagnostic, or prognostic marker in evaluating the risks of long term neurological consequences following TBI.

## Effective diagnosis and treatment of post-TBI neurological sequelae

Considering the significant risks incurred by TBI, it is clear that there is an imminent need for effective methods of early diagnosis, management, and monitoring of TBI patients to curtail the incidence of post-TBI neurological sequelae. At this time, diagnosis of TBI is based primarily on patient provided history and clinical observations [165–167]. Several clinical workflows have been developed for evaluation of mTBI, which is the most prevalent form of clinical TBI, including the Sport Concussion Assessment Tool and Military Acute Concussion Evaluation; however, these assessments are designed for use shortly following injury and, as such, rapidly diminish in sensitivity with delayed evaluation [168]. As well, the Glasgow Coma Scale has been in use for decades and allows for both rapid and consistent communication of patient condition [169]; nevertheless, the currently accepted threshold score of 13 may not be adequate to exclude visible abnormalities on computed tomography imaging that require neurosurgical intervention [170]. Due to these shortcomings in current diagnostic methodologies, civilian and military work groups have recommended the development of fluid or imaging based biomarkers for identification of mTBI [166, 168].

Several compounds and proteins have been suggested to serve as fluid biomarkers including glial fibrillary acidic protein (GFAP), calcium binding protein S100B, and tau protein [171]. In most cases, presence of these biomarkers is partially indicative of blood brain barrier disruption as they are typically confined within the central nervous system [171]. These proteins have been shown to be acutely elevated following TBI in human patients [172–174], but currently face challenges of low specificity [175, 176], poor correlation with development of post-concussive symptoms [177], and poor correlation with imaging abnormalities [178, 179].

Given the key role of oxidative stress and neuroinflammation in secondary neuronal injury and neurodegeneration, it is likely that the products of these processes may also serve as suitable biomarkers. As previously discussed, plasma levels of several oxidative stress and inflammation related markers have been observed to be elevated in serum up to 42 days after multiple blast injuries [50] and as early as 1 day following a single injury [53]. Furthermore, lipid peroxidation products, such as acrolein and 4-hydroxynonenal, have also been shown to be involved not only in TBI secondary injury [50, 53], but also in other modes of neuronal insult such as spinal cord injury [51, 180] and ischemia-reperfusion injury [181]. Given that these peroxidation products are not only indicative of damage, but also capable of causing modification of biomacromolecules, it is possible that measured elevations may be indicative not only of present damage, but also of continued secondary injury [49, 52, 182, 183]. As such, alleviation of oxidative stress could serve as a viable prophylactic strategy to diminish the risk of post TBI neurodegeneration. Direct supplementation with endogenous antioxidants, such as glutathione and superoxide dismutase, has not shown significant benefits as they do not easily cross the blood brain barrier [184–186]. However, the glutathione precursor N-acetylcysteine has shown some acute benefits in both animal and human studies [55, 187]. In addition, targeting of downstream components of the oxidative cascade, such as reactive aldehydes, has been suggested as a potential strategy due to the more extended half-lives of these compounds when compared to ROS [180, 184, 185]. However, despite extended elevation of inflammatory and oxidative byproducts, trials of antioxidant therapies have typically favored acute treatment, often within hours of the traumatic event, suggesting that acute treatment and monitoring may be more appropriate [184].

Considering the crucial role of post-TBI oxidative stress in the development and progression of chronic neurological diseases, detection and therapeutic targeting of this process appears to be a promising strategy for assessment, treatment, and monitoring of neurodegeneration risk post TBI. Given their connection to oxidative stress, inflammatory markers and lipid peroxidation byproducts could serve as surrogate biofluid markers. In addition, antioxidant treatment strategies can help neutralize perpetuation of cellular and molecular damage and diminish risks of long term neurological sequelae.

## Conclusion

Despite the prevalence of TBI in both civilian and military populations and the significant neurological sequelae incurred by such injuries, diagnosis and treatment of TBI remains poorly understood. Furthermore, the causative factors linking TBI to neurodegenerative diseases, such as AD, PD, ALS, and CTE, have not been fully elucidated. Several processes, including oxidative stress and neuroinflammation, have been found to be common between TBI secondary injury and several neurodegenerative diseases. In particular, oxidative stress appears to be the key mechanism linking neuroinflammation and glutamatergic excitotoxicity in both TBI and neurodegeneration. As such, it is probable that the oxidative cascade induced by TBI initiates and subsequently propagates the characteristic pathologies of neurodegeneration via oxidation or carbonylation of key proteins.

Due to the high prevalence of TBI and neurodegenerative diseases, the development of new effective strategies for early diagnosis and treatment for TBI is imperative. Given the key role that oxidative stress plays in linking secondary injury and neurodegeneration, detection of ROS and key byproducts could serve as a novel method for identification and monitoring of potential cellular damage. Furthermore, these reactive species may serve as a viable therapeutic target for reduction of long term neurodegeneration risk following TBI, having the potential to reduce the disability and death, and improve the quality of life in the long term of the civilian and military populations that suffer of TBI.

## Abbreviations

AD: Alzheimer’s disease
ALS: Amyotrophic lateral sclerosis
APP: Amyloid precursor protein
Aβ: Amyloid beta
CNS: Central nervous system
CTE: Chronic traumatic encephalopathy
GLT-1: Glutamate transporter 1
mTBI: Mild traumatic brain injury
PD: Parkinson’s disease
ROS: Reactive Oxygen Species
SOD1: Superoxide dismutase
TBI: Traumatic brain injury
